# Supplementary material for: Origin, specification and differentiation of a rare supporting-like lineage in the developing mouse gonad
Source: Sci Adv. 2022 May 25;8(21):eabm0972. doi: 10.1126/sciadv.abm0972 (PMC10942771; doi:10.1126/sciadv.abm0972)
Supplement: Supplementary file 1 — Supplementary Materials and Methods Figs. S1 to S8 Tables S1 and S2 References [file sciadv.abm0972_sm.pdf]

Science Advances
AAAS

Supplementary Materials for

Origin, specification and differentiation of a rare supporting-like lineage in the developing mouse gonad

Chloé Mayère *et al*.

Corresponding author: Serge Nef, serge.nef@unige.ch

Sci. Adv. **8**, eabm0972 (2022)
DOI: 10.1126/sciadv.abm0972

The PDF file includes:

Supplementary Materials and Methods
Figs. S1 to S8
Tables S1 and S2
Legends for data S1 to S5
References

Other Supplementary Material for this manuscript includes the following:

Data files S1 to S5

Supplementary Material & Methods

Whole-mount *in situ* hybridization

Whole-mount *in situ* hybridization (WISH) analysis of embryonic gonads and probes for *Sox9* and *Wnt4* have been previously described (*60*). At least three independent biological samples from XX and XY embryos were analyzed for each gene.

Inactivation of *Rar* coding genes

To inactivate *Rar* coding genes, mice bearing *lox*P-flanked (L2) alleles of *Rara* and *Rarg* and null (L-) alleles of *Rarb* were crossed with mice bearing the ubiquitously expressed, tamoxifen-inducible, Cre/ERT^2 recombinase. Noon of the day of a vaginal plug was taken as 0.5-day embryonic development (E0.5). To activate Cre/ERT^2 in embryos, one dose of tamoxifen (130 mg/kg body weight) was administered to the pregnant females at E10.5 by oral gavage. Following collection at E14.5, the tail of each embryo was sampled and used for genomic DNA extraction and genotyping as described (*20*). The embryos were then fixed overnight in cold 4% (w/v) paraformaldehyde (PFA) solution made in phosphate buffered saline (PBS). They were rinsed in PBS, placed in 70% (v/v) ethanol, and then embedded in paraffin. Consecutive, sagittal, 5µm-thick sections were cut throughout entire gonads. For FOXL2, PAX8 and SOX9 detection, demasking was performed for 1 hour at 95°C in 10 mM sodium citrate buffer at pH 6.0. Sections were rinsed in PBS, and then incubated for 16 hours at 4°C in a humidified chamber with the appropriate primary antibodies (see **Table S2**) diluted in PBS containing 0.1% (v/v) Tween 20 (PBST). After rinsing in PBST (3 times for 3 minutes each), sections were incubated for 45 minutes at 20°C in a humidified chamber with Cy3-conjugated or Alexa Fluor 488-conjugated secondary antibodies (see Table below). Nuclei were counterstained with 4',6-diamidino-2-phenylindole (DAPI) diluted at 10 µg/ml in the mounting medium (Vectashield, Vector Laboratories). For RARG detection, ImmPRESS® Polymer Detection Kit MP-7800-15 was used according to the manufacturer's protocol (Vector Laboratories). *Rara* and *Rarb* ablations were assessed by PCR analysis of genomic DNA extracted from 5 sections made at the level of the gonad and obtained from each embryo.

Supplementary Figure S1

A

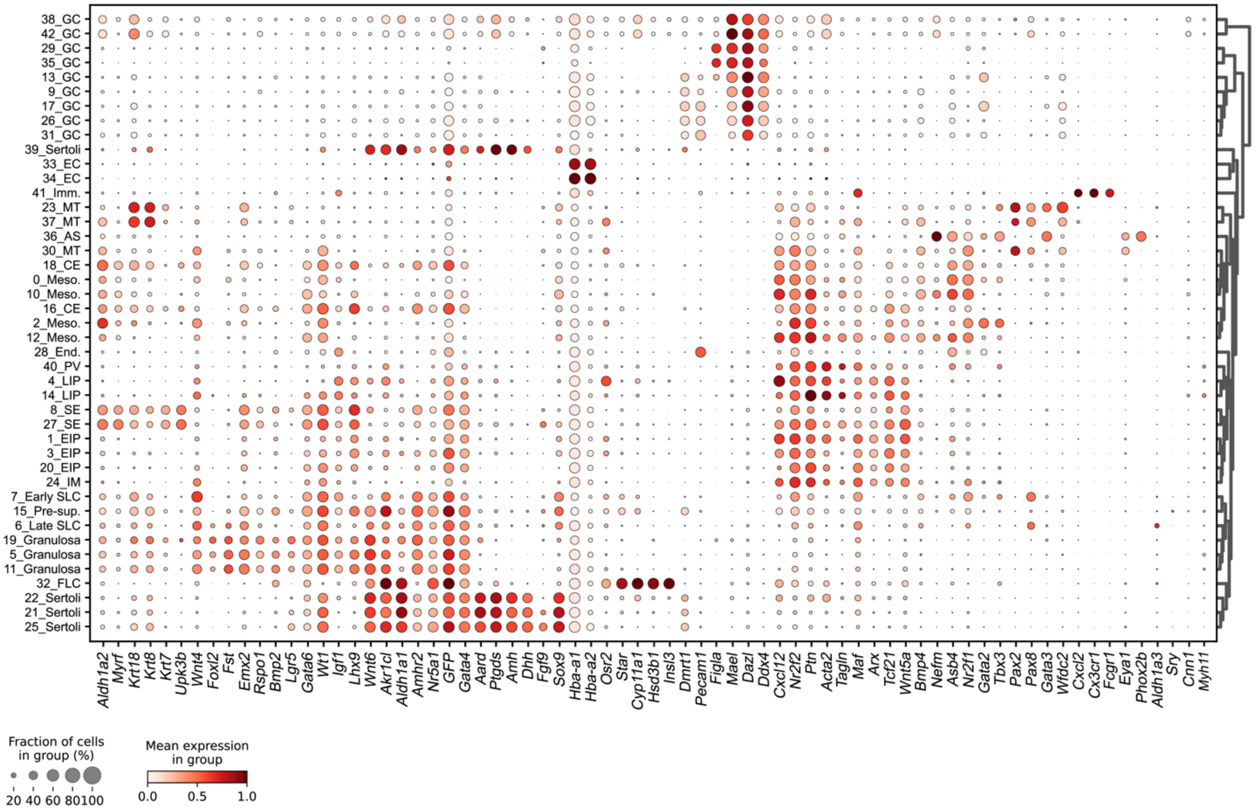

Fig. S1. Dotplot representation of the expression of marker genes in each Leiden cluster. The size of the node represents the percentage of cells in the cluster expressing the gene and the color indicates the average level of expression (log normalized counts). Clusters and genes are ordered by hierarchical clustering based on Spearman correlation. To facilitate the reading, cluster number are associated with inferred cell type annotation.

Supplementary Figure S2

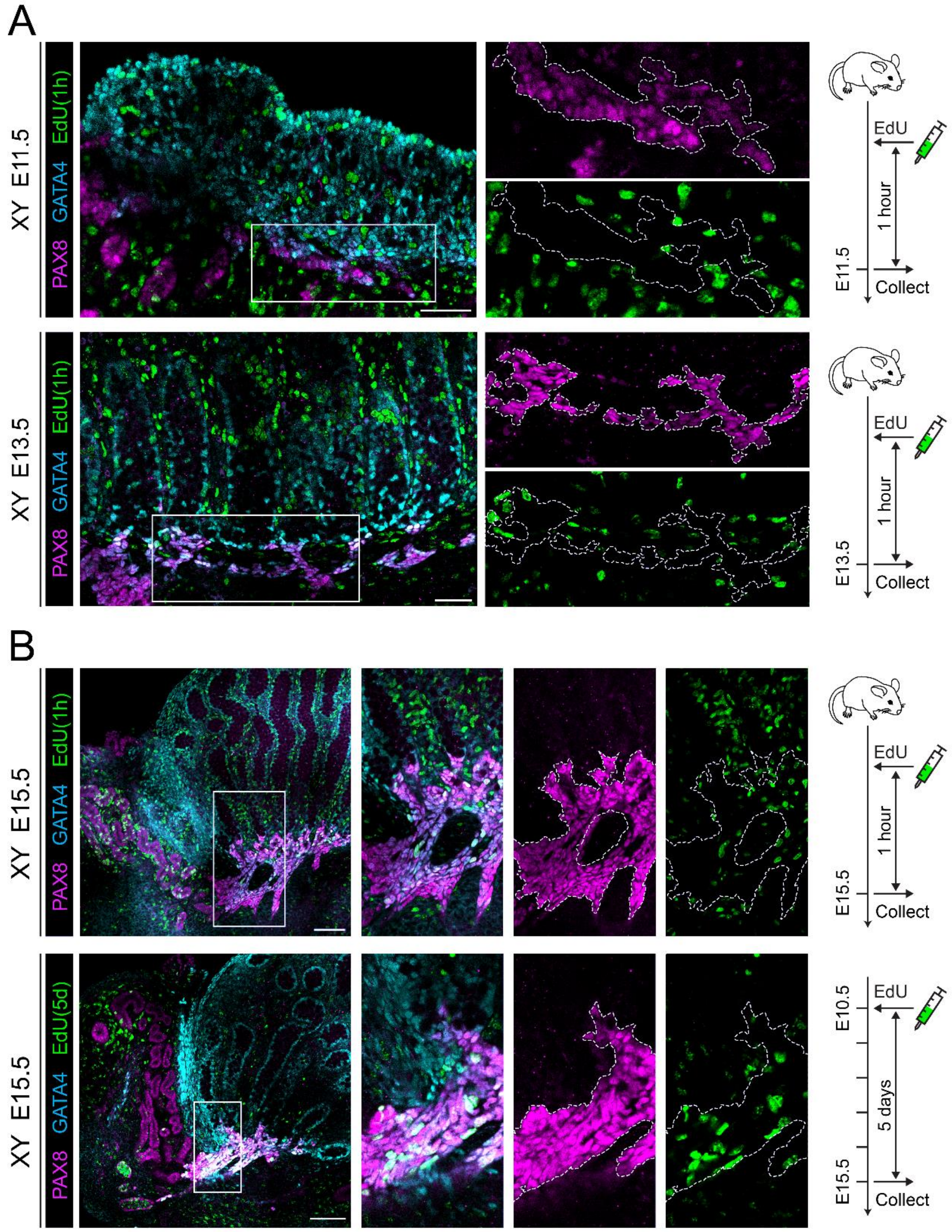

Fig. S2. EdU labeling of PAX8^{+} cells reveals that these cell cycle arrest in early population and a mixed population of cells in the rete testis at E15.5. (A-B) Wholemount immunofluorescence of gonads pulsed with EdU (green). Overlap of PAX8 (magenta) and GATA4 (cyan) was used to identify SLCs and the presumptive rete testis (dotted line). Boxes in merged images indicate regions shown as isolated channels on right. (A) XY gonads pulsed with EdU an hour before collection at E11.5 or E13.5. (B) XY gonads pulsed with EdU either 1 hour or 5 days before collection at E15.5. Scale bar = 50 µm.

Supplementary Figure S3

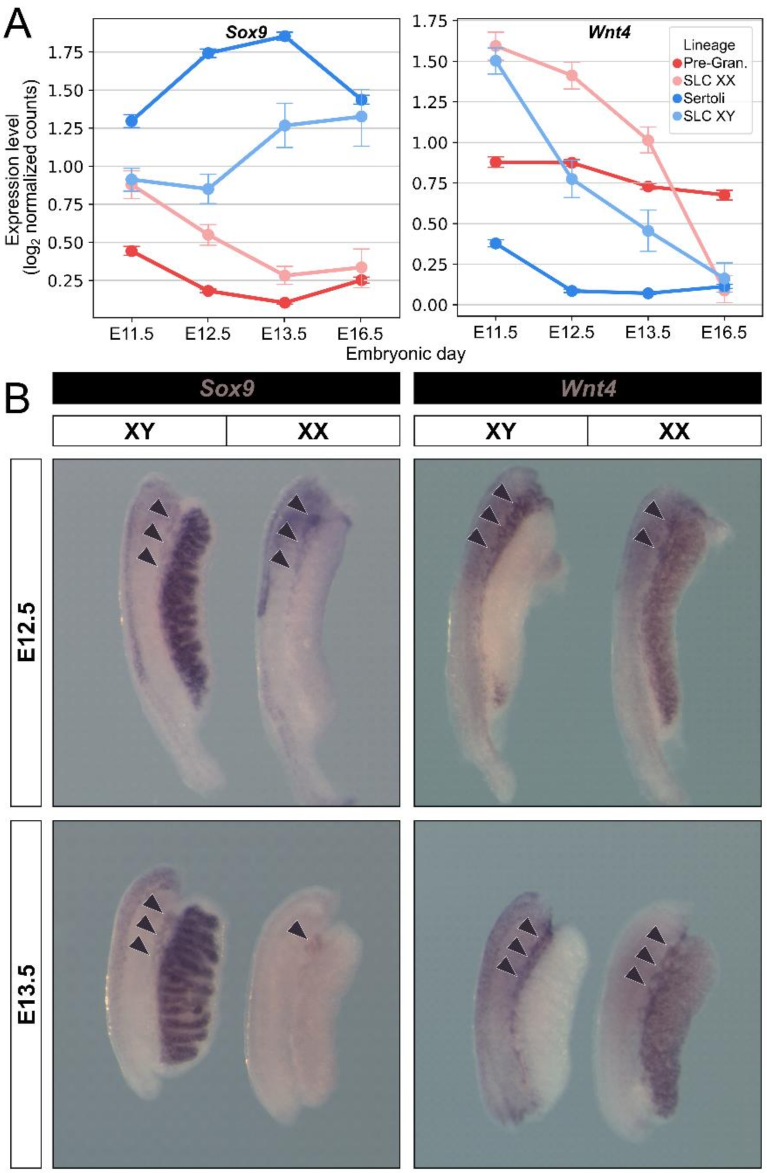

Fig. S3. ***Sox9* and *Wnt4* are both express in SLC of the rete testis and rete ovarii.** (A) Point plot representation of the expression of *Wnt4* and *Sox9* at the different developmental time points in both SLCs and supporting cells. Data were extracted from scRNA-seq analysis of XX and XY embryos at E11.5, E12.5, E13.5 and E16.5. (B) Whole-mount in-situ hybridization analysis of *Sox9* and *Wnt4* in XX and XY gonads at E12.5 and E13.5. *Wnt4* and *Sox9* transcripts were both detected in the rete testis and rete ovarii region (arrowheads).

Supplementary Figure S4

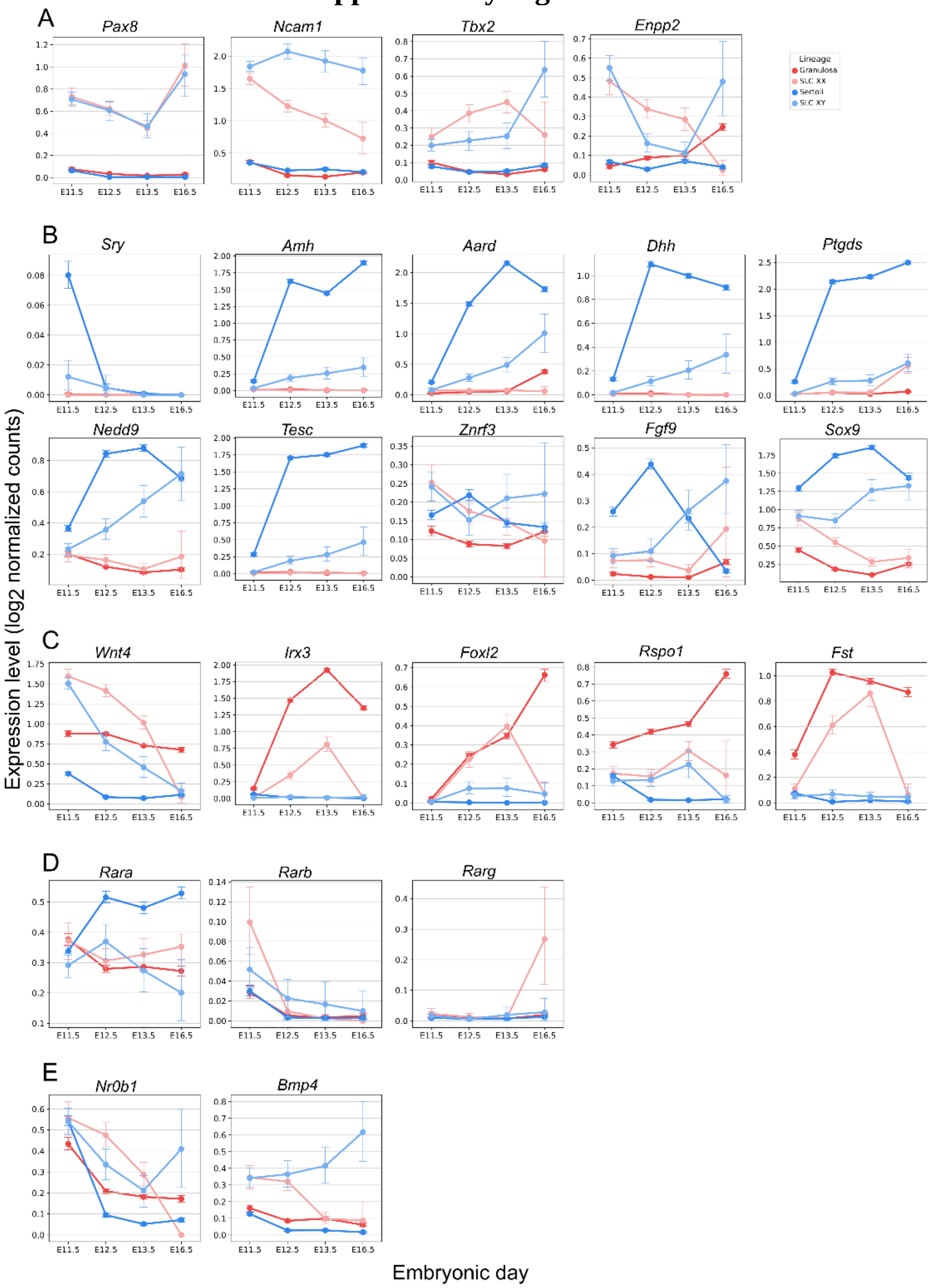

Fig. S4. Expression profiles of selected genes in SLC and supporting cells. Point plot representation of the expression of selected marker genes at the different developmental time points in both SLCs and supporting cells. Genes were classified according to their expression profiles or the relevance for a signaling pathway: (A) SLC, (B) Sertoli cells, (C) granulosa cells, (D) retinoic acid signaling and *Nr0b1* and *Bmp4* (E) miscellaneous.

Supplementary Figure S5

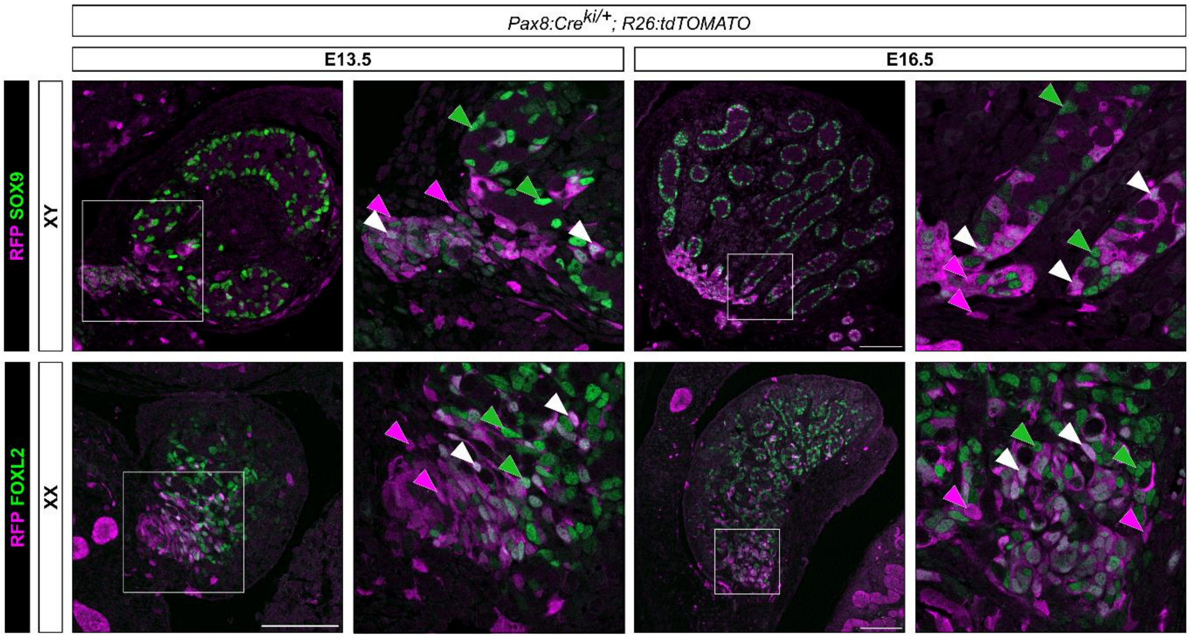

Fig. S5. SLC progenitors are multipotent and contribute to both rete structures and supporting cell lineage. Co-immunofluorescence for RFP/SOX9 and RFP/FOXL2 in XY and XX gonads of *Pax8:Cre;Rosa26:tdTomato;Nr5a1:GFP* at E13.5 and E16.5. Magenta arrowheads indicate RFP^+ cells, white arrowheads indicate $RFP^+/SOX9^+$ or $RFP^+/FOXL2^+$ cells, green arrowheads indicate $SOX9^+$ or $FOXL2^+$ cells. Presence of RFP+ cells is highest near the rete. $RFP^+/SOX9^+$ cells are present in testis cords at E13.5 and E16.5. Similarly, $RFP^+/FOXL2^+$ cells are present in the ovary at E13.5 and E16.5. Scale bar 100 µm, insets are 50 µm wide.

Supplementary Figure S6

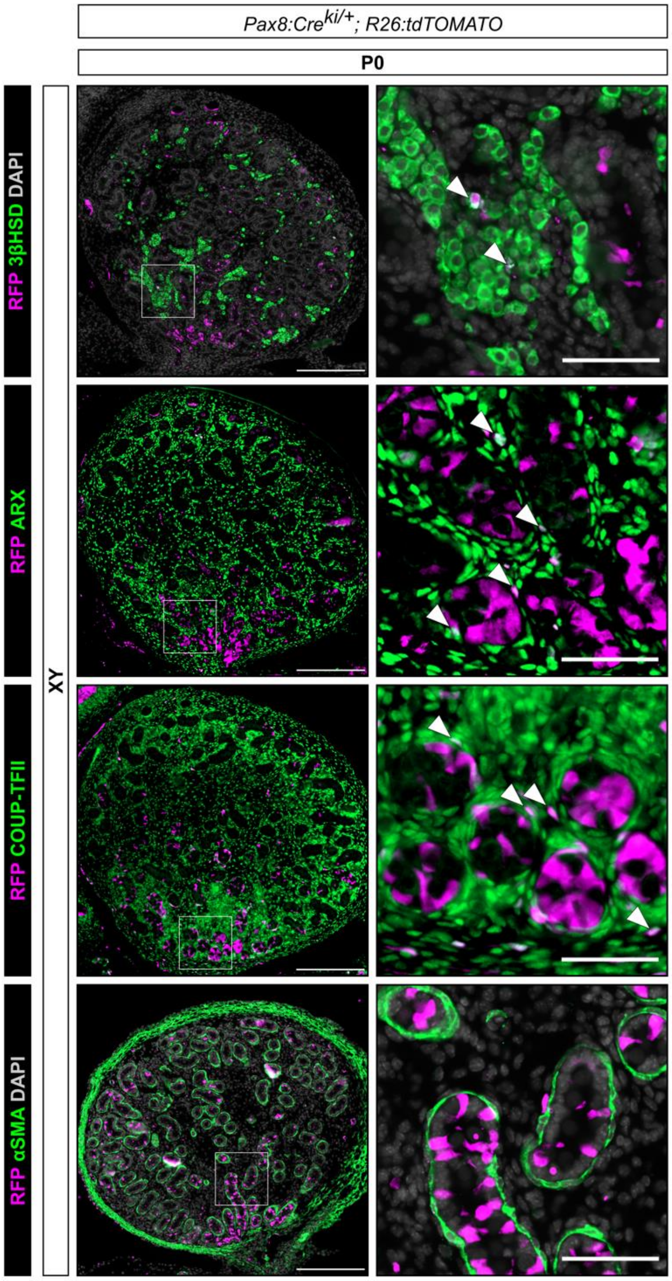

Fig. S6. SLCs rarely contribute to the population of Leydig cells, interstitial progenitors and peritubular myoid cells in XY gonads. Co-immunofluorescence of XY gonads of *Pax8:Cre;Rosa26:tdTomato;Nr5a1:GFP* mice at P0 for RFP and 3βHSD, a marker of the Leydig cells, RFP and ARX or COUP-TFII, two markers of interstitial progenitors, and RFP and αSMA, a marker of the peritubular myoid cells. Boxes indicate regions shown on the right. White arrowheads indicate co-labeled cells. Scale bars 200 µm and 50 µm.

Supplementary Figure S7

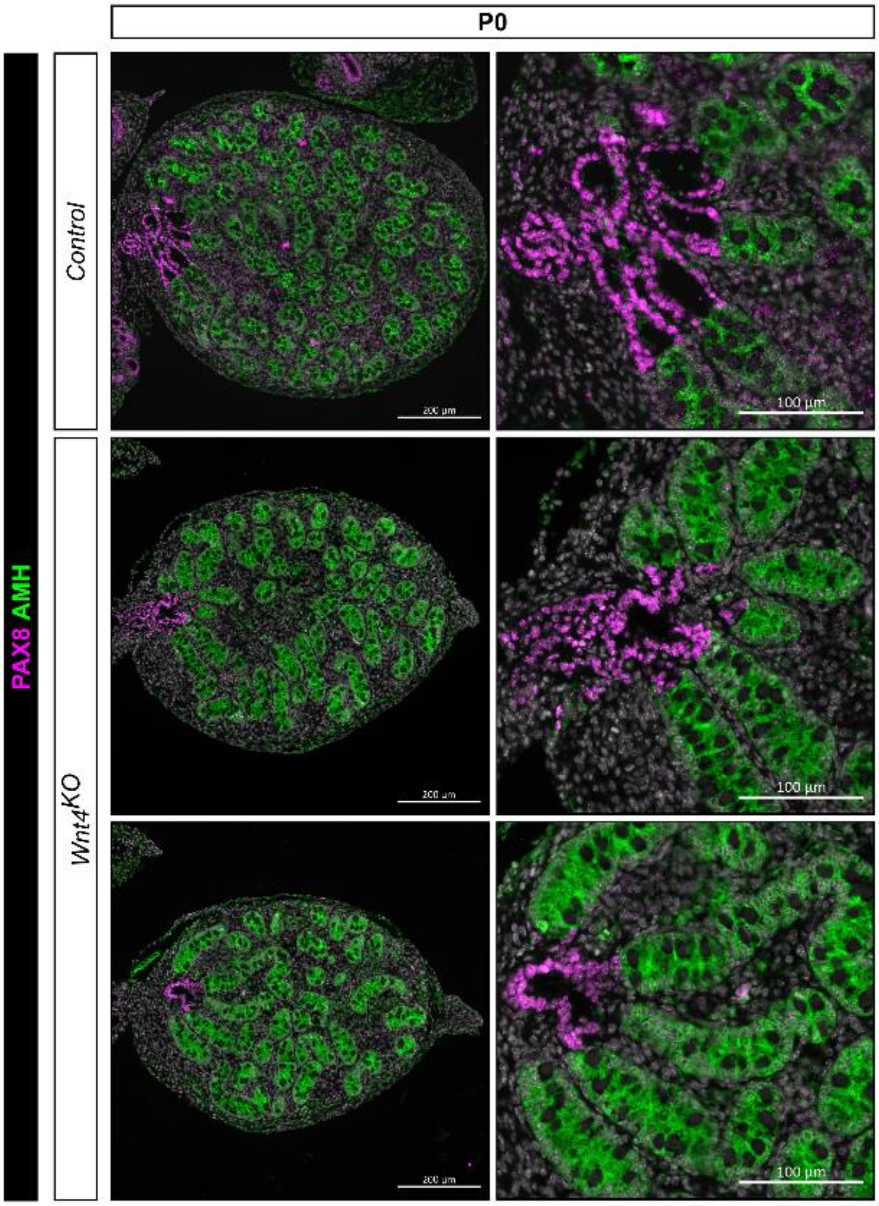

Fig. S7: *Wnt4* is required for normal rete testis development. Representative double immunofluorescence against PAX8 and AMH in control (*Wnt4KO*$^{+/+}$) or Wnt4KO (*Wnt4:KO*$^{-/-}$) XY embryos at P0. Boxes indicate regions shown on the right magnifying the rete testis and adjacent testis cords. The rete testis appears to be hypoplastic and connects fewer testis cords than in control testes. Scale bars 200 µm and 100 µm in insets.

Supplementary Figure S8

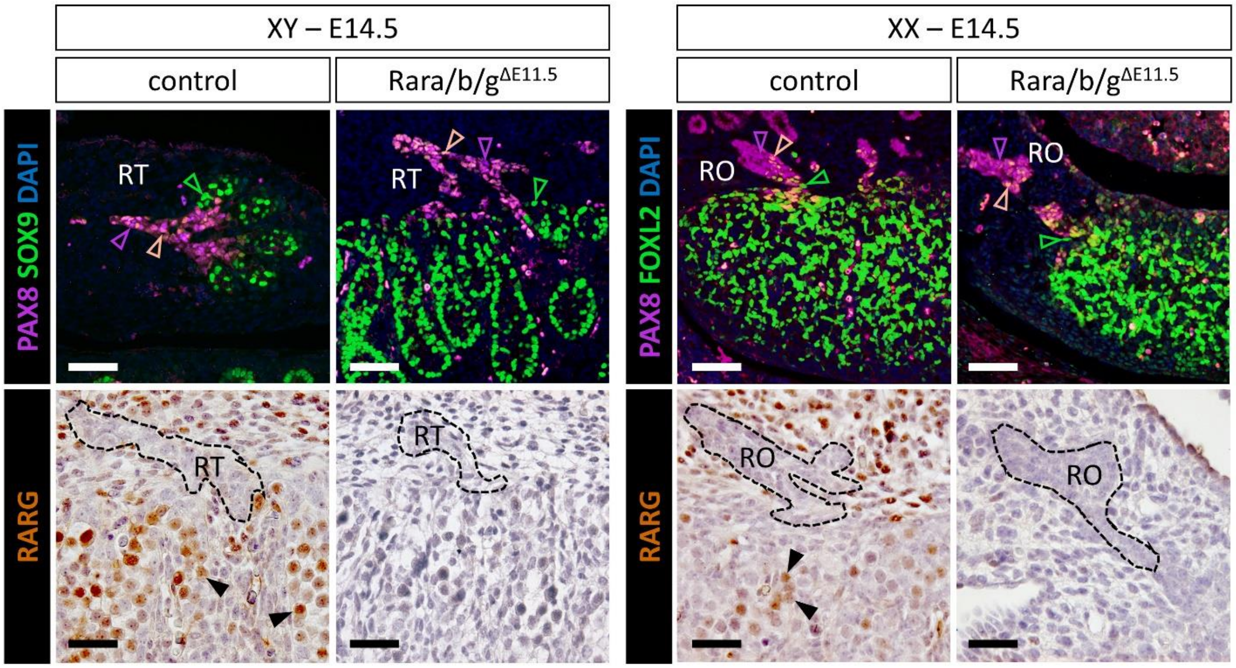

Fig. S8. Ablation of all *Rar*-coding genes from E11.5 onwards does not impair formation and differentiation of rete testis/ovarii cells. Upper left panel: immuno-detection of PAX8 (magenta signal), SOX9 (green signal) in control and mutant (Rara/b/g$^{\Delta E11.5}$) male (XY) embryos at E14.5. Upper right panel: immuno-detection of PAX8 (magenta signal), FOXL2 (green signal) in control and mutant (Rara/b/g$^{\Delta E11.5}$) female (XX) embryos at E14.5. Lower left panel: immuno-detection of RARG (in brown) in control and mutant (Rara/b/g$^{\Delta E11.5}$) male (XY) embryos at E14.5. Nuclei were counterstained with DAPI (blue signal). Note that PAX8^{+}/SOX9^{+} and PAX8^{+}/FOXL2^{+} cells appear beige. Lower right panel: immuno-detection of RARG (in brown) in control and mutant (Rara/b/g$^{\Delta E11.5}$) female (XX) embryos at E14.5. Note that (i) in the control situation, RARG is detected mainly in germ cells and mesonephric mesenchymal cells, but not in rete testis/ovarii cells; (ii) in Rara/b/g$^{\Delta E11.5}$ embryos, RARG is efficiently lost in virtually all cell-types, as anticipated (see (Vernet, et al., 2020)). Legend: RO, rete ovarii; RT, rete testis; XX, female fetus; XY, male fetus; the dotted lines delineate the rete testis/ovarii; magenta, beige and green open arrowheads point to PAX8^{+}, PAX8^{+}/SOX9^{+} or PAX8^{+}/FOXL2^{+}, and SOX9^{+} or FOXL2^{+} cells, respectively; black filled arrowheads point to RARG^{+} germ cells. Scale bars is 50 µm.

Table S1

Supplementary Table 1: Relative abundance of cell types during the process of sex determination (in percentage)

Developmental stage	E10.5		E11.5		E12.5		E13.5		E16.5	
Genetic sex	XX	XY	XX	XY	XX	XY	XX	XY	XX	XY
Cell type										
Germ cells	0,2	0,8	5,6	5,8	27,2	16,6	27,7	24,3	34,0	6,0
Coelomic epithelium	10,4	20,3	11,3	17,9	0,1	0,1	0,0	0,0	0,0	0,0
Surface epithelium	0,0	0,0	0,2	0,2	9,0	12,1	15,9	7,1	0,6	2,5
Pre-supporting	0,0	0,0	15,3	16,7	0,0	0,0	0,0	0,0	0,0	0,0
Sertoli	0,0	0,0	0,0	0,6	0,6	17,1	0,0	14,4	0,0	22,1
Granulosa	0,0	0,0	0,1	0,0	35,9	0,1	35,1	0,0	35,4	0,0
Early supporting-like	0,0	0,0	2,7	2,7	0,0	0,0	0,0	0,0	0,0	0,0
Late supporting-like	0,0	0,0	0,0	0,0	3,6	1,6	2,0	0,7	0,1	0,3
Fetal Leydig	0,0	0,0	0,0	0,0	0,0	0,1	0,0	4,1	0,0	4,8
Early interst. progenitors	0,0	0,0	0,1	0,2	8,7	39,2	15,1	38,4	0,1	0,2
Late interst. progenitors	0,0	0,0	0,0	0,0	0,0	0,0	0,0	0,2	24,7	57,6
Invading Meson.	0,0	0,0	0,1	0,0	7,8	4,8	0,2	4,3	0,0	0,1
Perivascular	0,0	0,0	0,0	0,0	0,0	0,1	0,0	1,1	0,7	1,0
Mesonephric tubules	20,0	16,2	7,6	5,8	1,1	0,3	0,0	0,1	0,0	0,0
Mesonephric mesenchyme	64,5	56,7	54,8	47,3	0,2	0,9	0,0	0,0	0,0	0,0
Endothelium	0,7	1,6	1,2	1,3	1,9	4,0	1,3	2,1	0,6	0,7
Immune	0,3	0,2	0,3	0,5	0,3	0,3	0,2	0,3	0,5	0,5
Adrenosympatic	3,7	3,7	0,4	0,4	0,0	0,1	0,0	0,0	0,0	0,0
Erythrocytes	0,2	0,5	0,3	0,6	3,7	2,8	2,3	3,0	3,2	4,1

Table S2

Supplementary table 2: detailed information on the antibodies used in this study

**Antigen**	**Species**	**Reference**	**Source**	**Dilution**	**Secondary antibody or Detection**
FOXL2	Goat	ab5096	Abcam	1/200	Alexa Fluor 488-conjugated donkey anti-goat
SOX9	Rabbit	AB5535	Millipore	1/1000	Alexa Fluor 488-conjugated donkey anti-rabbit
PAX8	Mouse	ab53490	Abcam	1/10	Cy3-conjugated donkey anti-mouse
RARG	Rabbit	8965S	Cell Signaling	1/200	ImmPRESS® Polymer Detection Kit MP-7800-15

Legends for data S1-S4

Data S1: Differentially expressed genes between XY SLCs and supporting cells at early, late and all stages - associated GO biological process terms.
Data S2: Differentially expressed genes between XX SLCs and supporting cells at early, late and all stages. - associated GO biological process terms.
Data S3: Differentially expressed genes between cells from subclusters 5,1 and 5,0.
Data S4: Genes selected by lasso modeling for granulosa and Sertoli cells and associated weights
Data S5: Differentially expressed genes between XX and XY SLCs at early, late and all stages - associated GO biological process terms.

REFERENCES AND NOTES

1. E. Rotgers, A. Jorgensen, H.-H. Yao, At the crossroads of fate—Somatic cell lineage specification in the fetal gonad. *Endocr. Rev.* **39**, 739–759 (2018).

2. Y. C. Hu, L. M. Okumura, D. C. Page, Gata4 is required for formation of the genital ridge in mice. *PLOS Genet.* **9**, e1003629 (2013).

3. Y. Ikeda, W. H. Shen, H. A. Ingraham, K. L. Parker, Developmental expression of mouse steroidogenic factor-1, an essential regulator of the steroid hydroxylases. *Mol. Endocrinol.* **8**, 654–662 (1994).

4. J. A. Kreidberg, H. Sariola, J. M. Loring, M. Maeda, J. Pelletier, D. Housman, R. Jaenisch, WT-1 is required for early kidney development. *Cell* **74**, 679–691 (1993).

5. J. Karl, B. Capel, Sertoli cells of the mouse testis originate from the coelomic epithelium. *Dev. Biol.* **203**, 323–333 (1998).

6. I. Stevant, F. Kühne, A. Greenfield, M.-C. Chaboissier, E. T. Dermitzakis, S. Nef, Dissecting cell lineage specification and sex fate determination in gonadal somatic cells using single-cell transcriptomics. *Cell Rep.* **26**, 3272–3283.e3 (2019).

7. I. Stevant, Y. Neirijnck, C. Borel, J. Escoffier, L. B. Smith, S. E. Antonarakis, E. T. Dermitzakis, S. Nef, Deciphering cell lineage specification during male sex determination with single-cell RNA sequencing. *Cell Rep.* **22**, 1589–1599 (2018).

8. S. Nef, I. Stevant, A. Greenfield, Characterizing the bipotential mammalian gonad. *Curr. Top. Dev. Biol.* **134**, 167–194 (2019).

9. Y. T. Lin, L. Barske, T. DeFalco, B. Capel, Numb regulates somatic cell lineage commitment during early gonadogenesis in mice. *Development* **144**, 1607–1618 (2017).

10. I. B. Barsoum, H. H. Yao, Fetal Leydig cells: Progenitor cell maintenance and differentiation. *J. Androl.* **31**, 11–15 (2010).

11. J. Brennan, C. Tilmann, B. Capel, Pdgfr-α mediates testis cord organization and fetal Leydig cell development in the XY gonad. *Genes Dev.* **17**, 800–810 (2003).

12. M. Inoue, Y. Shima, K. Miyabayashi, K. Tokunaga, T. Sato, T. Baba, Y. Ohkawa, H. Akiyama, M. Suyama, K. I. Morohashi, Isolation and characterization of fetal Leydig progenitor cells of male mice. *Endocrinology* **157**, 1222–1233 (2016).

13. C. Liu, K. Rodriguez, H. H. Yao, Mapping lineage progression of somatic progenitor cells in the mouse fetal testis. *Development* **143**, 3700–3710 (2016).

14. K. Miyabayashi, Y. Katoh-Fukui, H. Ogawa, T. Baba, Y. Shima, N. Sugiyama, K. Kitamura, K. I. Morohashi, Aristaless related homeobox gene, Arx, is implicated in mouse fetal Leydig cell differentiation possibly through expressing in the progenitor cells. *PLOS ONE* **8**, e68050 (2013).

15. A. Y. Kulibin, E. A. Malolina, Formation of the rete testis during mouse embryonic development. *Dev. Dyn.* **249**, 1486–1499 (2020).

16. A. T. Major, M. A. Estermann, C. A. Smith, Anatomy, endocrine regulation and embryonic development of the rete testis. *Endocrinology* **162**, bqab046 (2021).

17. E. A. Malolina, A. Y. Kulibin, The rete testis harbors Sertoli-like cells capable of expressing DMRT1. *Reproduction (Cambridge, England)* **158**, 399–413 (2019).

18. T. Omotehara, X. Wu, M. Kuramasu, M. Itoh, Connection between seminiferous tubules and epididymal duct is originally induced before sex differentiation in a sex-independent manner. *Dev. Dyn.* **249**, 754–764 (2020).

19. J. McKey, D. N. Anbarci, C. Bunce, B. Capel, Integration of mouse ovary morphogenesis with developmental dynamics of the oviduct, ovarian ligaments, and rete ovarii. *bioRxiv preprint*, (2021).

20. J. G. Wenzel, S. Odend'hal, The mammalian rete ovarii: A literature review. *Cornell Vet.* **75**, 411–425 (1985).

21. A. G. Byskov, Does the rete ovarii act as a trigger for the onset of meiosis? *Nature* **252**, 396–397 (1974).

22. P. Smith, D. Wilhelm, R. J. Rodgers, Development of mammalian ovary. *J. Endocrinol.* **221**, R145–161 (2014).

23. V. A. Traag, L. Waltman, N. J. van Eck, From Louvain to Leiden: Guaranteeing well-connected communities. *Sci. Rep.* **9**, 5233 (2019).

24. P. S. Western, D. C. Miles, J. A. van den Bergen, M. Burton, A. H. Sinclair, Dynamic regulation of mitotic arrest in fetal male germ cells. *Stem Cells* **26**, 339–347 (2008).

25. C. Bunce, J. McKey, B. Capel, Concerted morphogenesis of genital ridges and nephric ducts in the mouse captured through whole-embryo imaging. *Development* **148**, (2021).

26. M. Bouchard, A. Souabni, M. Mandler, A. Neubuser, M. Busslinger, Nephric lineage specification by Pax2 and Pax8. *Genes Dev.* **16**, 2958–2970 (2002).

27. M. Plass, J. Solana, F. A. Wolf, S. Ayoub, A. Misios, P. Glažar, B. Obermayer, F. J. Theis, C. Kocks, N. Rajewsky, Cell type atlas and lineage tree of a whole complex animal by single-cell transcriptomics. *Science* **360**, (2018).

28. F. A. Wolf, F. K. Hamey, M. Plass, J. Solana, J. S. Dahlin, B. Göttgens, N. Rajewsky, L. Simon, F. J. Theis, PAGA: Graph abstraction reconciles clustering with trajectory inference through a topology preserving map of single cells. *Genome Biol.* **20**, 59 (2019).

29. R. Satija, J. A. Farrell, D. Gennert, A. F. Schier, A. Regev, Spatial reconstruction of single-cell gene expression data. *Nat. Biotechnol.* **33**, 495–502 (2015).

30. G. Finak, A. McDavid, M. Yajima, J. Deng, V. Gersuk, A. K. Shalek, C. K. Slichter, H. W. Miller, M. J. McElrath, M. Prlic, P. S. Linsley, R. Gottardo, MAST: A flexible statistical framework for assessing transcriptional changes and characterizing heterogeneity in single-cell RNA sequencing data. *Genome Biol.* **16**, 278 (2015).

31. R. H. Rastetter, P. Bernard, J. S. Palmer, A. A. Chassot, H. Chen, P. S. Western, R. G. Ramsay, M. C. Chaboissier, D. Wilhelm, Marker genes identify three somatic cell types in the fetal mouse ovary. *Dev. Biol.* **394**, 242–252 (2014).

32. W. Zheng, H. Zhang, N. Gorre, S. Risal, Y. Shen, K. Liu, Two classes of ovarian primordial follicles exhibit distinct developmental dynamics and physiological functions. *Hum. Mol. Genet.* **23**, 920–928 (2014).

33. W. Niu, A. C. Spradling, Two distinct pathways of pregranulosa cell differentiation support follicle formation in the mouse ovary. *Proc. Natl. Acad. Sci. U.S.A.* **117**, 20015–20026 (2020).

34. M. Bouchard, A. Souabni, M. Busslinger, Tissue-specific expression of cre recombinase from the Pax8 locus. *Genesis* **38**, 105–109 (2004).

35. N. R. Stallings, N. A. Hanley, G. Majdic, L. Zhao, M. Bakke, K. L. Parker, Development of a transgenic green fluorescent protein lineage marker for steroidogenic factor 1. *Endocr. Res.* **28**, 497–504 (2002).

36. K. Jeays-Ward, M. Dandonneau, A. Swain, Wnt4 is required for proper male as well as female sexual development. *Dev. Biol.* **276**, 431–440 (2004).

37. K. Stark, S. Vainio, G. Vassileva, A. P. McMahon, Epithelial transformation of metanephric mesenchyme in the developing kidney regulated by Wnt-4. *Nature* **372**, 679–683 (1994).

38. F. J. Barrionuevo, M. Burgos, G. Scherer, R. Jimenez, Genes promoting and disturbing testis development. *Histol. Histopathol.* **27**, 1361–1383 (2012).

39. B. Jeffs, J. J. Meeks, M. Ito, F. A. Martinson, M. M. Matzuk, J. L. Jameson, L. D. Russell, Blockage of the rete testis and efferent ductules by ectopic Sertoli and Leydig cells causes infertility in Dax1-deficient male mice. *Endocrinology* **142**, 4486–4495 (2001).

40. J. Bowles, C. W. Feng, J. Ineson, K. Miles, C. M. Spiller, V. R. Harley, A. H. Sinclair, P. Koopman, Retinoic acid antagonizes testis development in mice. *Cell Rep.* **24**, 1330–1341 (2018).

41. F. Nagl, K. Schönhofer, B. Seidler, J. Mages, H. D. Allescher, R. M. Schmid, G. Schneider, D. Saur, Retinoic acid-induced nNOS expression depends on a novel PI3K/Akt/DAX1 pathway in human TGW-nu-I neuroblastoma cells. *Am. J. Physiol. Cell Physiol.* **297**, C1146–C1156 (2009).

42. M. Ito, R. Yu, J. L. Jameson, DAX-1 inhibits SF-1-mediated transactivation via a carboxy-terminal domain that is deleted in adrenal hypoplasia congenita. *Mol. Cell. Biol.* **17**, 1476–1483 (1997).

43. M. W. Nachtigal, Y. Hirokawa, D. L. Enyeart-VanHouten, J. N. Flanagan, G. D. Hammer, H. A. Ingraham, Wilms' tumor 1 and Dax-1 modulate the orphan nuclear receptor SF-1 in sex-specific gene expression. *Cell* **93**, 445–454 (1998).

44. S. Y. Park, J. J. Meeks, G. Raverot, L. E. Pfaff, J. Weiss, G. D. Hammer, J. L. Jameson, Nuclear receptors Sf1 and Dax1 function cooperatively to mediate somatic cell differentiation during testis development. *Development* **132**, 2415–2423 (2005).

45. N. Vernet, D. Condrea, C. Mayere, B. Féret, M. Klopfenstein, W. Magnant, V. Alunni, M. Teletin, S. Souali-Crespo, S. Nef, M. Mark, N. B. Ghyselinck, Meiosis occurs normally in the fetal ovary of mice lacking all retinoic acid receptors. *Sci Adv* **6**, eaaz1139 (2020).

46. A. A. Chassot, F. Ranc, E. P. Gregoire, H. L. Roepers-Gajadien, M. M. Taketo, G. Camerino, D. G. de Rooij, A. Schedl, M. C. Chaboissier, Activation of beta-catenin signaling by Rspo1 controls differentiation of the mammalian ovary. *Hum. Mol. Genet.* **17**, 1264–1277 (2008).

47. B. Nicol, H. H. Yao, Gonadal identity in the absence of pro-testis factor SOX9 and pro-ovary factor beta-catenin in mice. *Biol. Reprod.* **93**, 35 (2015).

48. S. Vainio, M. Heikkila, A. Kispert, N. Chin, A. P. McMahon, Female development in mammals is regulated by Wnt-4 signalling. *Nature* **397**, 405–409. (1999).

49. B. Lustig, B. Jerchow, M. Sachs, S. Weiler, T. Pietsch, U. Karsten, M. van de Wetering, H. Clevers, P. M. Schlag, W. Birchmeier, J. Behrens, Negative feedback loop of Wnt signaling through upregulation of conductin/axin2 in colorectal and liver tumors. *Mol. Cell. Biol.* **22**, 1184–1193 (2002).

50. L. Madisen, T. A. Zwingman, S. M. Sunkin, S. W. Oh, H. A. Zariwala, H. Gu, L. L. Ng, R. D. Palmiter, M. J. Hawrylycz, A. R. Jones, E. S. Lein, H. Zeng, A robust and high-throughput Cre reporting and characterization system for the whole mouse brain. *Nat. Neurosci.* **13**, 133–140 (2010).

51. F. Tang, N. Richardson, A. Albina, M. C. Chaboissier, A. Perea-Gomez, Mouse gonad development in the absence of the pro-ovary factor WNT4 and the pro-testis factor SOX9. *Cell* **9**, (2020).

52. L. McFarlane, V. Truong, J. S. Palmer, D. Wilhelm, Novel PCR assay for determining the genetic sex of mice. *Sex. Dev.* **7**, 207–211 (2013).

53. I. Stevant, S. Nef, Single cell transcriptome sequencing: A new approach for the study of mammalian sex determination. *Mol. Cell. Endocrinol.* **468**, 11–18 (2018).

54. S. Miyawaki, S. Kuroki, R. Maeda, N. Okashita, P. Koopman, M. Tachibana, The mouse Sry locus harbors a cryptic exon that is essential for male sex determination. *Science* **370**, 121–124 (2020).

55. K. Polanski, M. D. Young, Z. Miao, K. B. Meyer, S. A. Teichmann, J.-E. Park, BBKNN: Fast batch alignment of single cell transcriptomes. *Bioinformatics* **36**, 964–965 (2020).

56. J. H. Ward, Hierarchical grouping to optimize an objective function. *J. Am. Stat. Assoc.* **58**, 236–244 (1963).

57. J. C. Polanco, D. Wilhelm, T. L. Davidson, D. Knight, P. Koopman, Sox10 gain-of-function causes XX sex reversal in mice: Implications for human 22q-linked disorders of sex development. *Hum. Mol. Genet.* **19**, 506–516 (2010).

58. P. Bankhead, M. B. Loughrey, J. A. Fernández, Y. Dombrowski, D. G. McArt, P. D. Dunne, S. McQuaid, R. T. Gray, L. J. Murray, H. G. Coleman, J. A. James, M. Salto-Tellez, P. W. Hamilton, QuPath: Open source software for digital pathology image analysis. *Sci. Rep.* **7**, 16878 (2017).

59. R. Tomer, L. Ye, B. Hsueh, K. Deisseroth, Advanced CLARITY for rapid and high-resolution imaging of intact tissues. *Nat. Protoc.* **9**, 1682–1697 (2014).

60. N. Warr, P. Siggers, D. Bogani, R. Brixey, L. Pastorelli, L. Yates, C. H. Dean, S. Wells, W. Satoh, A. Shimono, A. Greenfield, Sfrp1 and Sfrp2 are required for normal male sexual development in mice. *Dev. Biol.* **326**, 273–284 (2009).
